# Mistaken Pregnancy

**Published:** 1875-04

**Authors:** 


In the *Medical Times* Dr. E. K. Lewis, of Kansas City, writes: On my arrival, I found a large German woman, apparently in active labor. I immediately made an examination; waited a few minutes for a pain, but no pain came. On placing my hand upon the abdomen, I was surprised to find very little tumefaction. On questioning the woman, I found she had been married twice, had borne two children by her first husband; none by her second; and had been married to him only twelve months. She also remarked, "I have never missed a month." "Then why do you think you are pregnant?" "Because I am eight inches larger around the waist than when I was married, and I am so large I

have been ashamed to be seen on the street for a month past, and often I have felt the movement of the child." Not believing her to be pregnant, (notwithstanding the baby-clothes, etc., she had lying around the room,) I gave her one-fourth grain of morphia, and left. The next day Professor Taylor and myself examined the woman thoroughly, and found the os normal; but in passing the sound the interior of the womb was quite sensitive, and a slight discharge of blood followed the withdrawal, but no child, no ovarian trouble, no fibroid, no polypus, and, in short, nothing that would point to any abnormal trouble whatever. It seems that since the woman's last marriage she had gained some thirty-five or forty pounds of flesh, and, her abdominal muscles being very much relaxed, the whole viscera have gravitated towards the pelvis, giving much the appearance of a pregnant woman.

